# Expanding the Arterivirus Host Spectrum: Olivier’s Shrew Virus 1, A Novel Arterivirus Discovered in African Giant Shrews

**DOI:** 10.1038/s41598-018-29560-x

**Published:** 2018-07-24

**Authors:** Bert Vanmechelen, Valentijn Vergote, Lies Laenen, Fara Raymond Koundouno, Joseph Akoi Bore, Jiro Wada, Jens H. Kuhn, Miles W. Carroll, Piet Maes

**Affiliations:** 1 KU Leuven, Department of Microbiology and Immunology, Laboratory of Clinical Virology, Rega Institute for Medical Research, Herestraat 49-Box 1040, BE3000 Leuven, Belgium; 2 University Julius Nyerere of Kankan, Conakry, Guinea; 3 Institut National de Santé Publique, Conakry, Guinea; 4 Integrated Research Facility at Fort Detrick, National Institute of Allergy and Infectious Diseases, National Institutes of Health, B-8200 Research Plaza, Fort Detrick, Frederick, Maryland 21702 USA; 5 Research & Development Institute, National Infections Service, Public Health England, Porton Down, Salisbury, United Kingdom

## Abstract

The family *Arteriviridae* harbors a rapidly expanding group of viruses known to infect a divergent group of mammals, including horses, pigs, possums, primates, and rodents. Hosts infected with arteriviruses present with a wide variety of (sub) clinical symptoms, depending on the virus causing the infection and the host being infected. In this study, we determined the complete genome sequences of three variants of a previously unknown virus found in Olivier’s shrews (*Crocidura olivieri guineensis*) sampled in Guinea. On the nucleotide level, the three genomes of this new virus, named Olivier’s shrew virus 1 (OSV-1), are 88–89% similar. The genome organization of OSV-1 is characteristic of all known arteriviruses, yet phylogenetic analysis groups OSV-1 separately from all currently established arterivirus lineages. Therefore, we postulate that OSV-1 represents a member of a novel arterivirus genus. The virus described here represents the first discovery of an arterivirus in members of the order Eulipotyphla, thereby greatly expanding the known host spectrum of arteriviruses.

## Introduction

Arteriviruses are a group of viruses assigned to the family *Arteriviridae*, one of four accepted families within the order *Nidovirales*^1^. Arteriviruses have positive-sense, single-stranded linear RNA genomes and produce enveloped spherical particles^2^. As of 2017, arteriviruses are known to infect equids (genus *Equartevirus*), pigs (genus *Porartevirus*), possums (genus *Dipartevirus*), nonhuman primates (genus *Simartevirus*), and rodents (genera *Porartevirus* and *Nesartevirus*)^1,3^. Several known arteriviruses are capable of causing overt, severe disease^2,3^. The equartevirus equine arteritis virus (EAV) causes mild disease in equids but can also cause severe respiratory distress, typically in foals, or lead to abortion in pregnant mares^4^. A comparable clinical presentation, reproductive failure or respiratory distress, is seen in neonatal pigs infected with the porarteviruses porcine reproductive and respiratory syndrome virus 1 and 2 (PRRSV-1/2)^5^. The dipartevirus wobbly possum disease virus (WPDV) causes an often fatal neurological syndrome in possums^6^, whereas several simarteviruses (e.g., Pebjah virus [PBJV], simian hemorrhagic encephalitis virus [SHEV], and simian hemorrhagic fever virus [SHFV]) can cause highly lethal hemorrhagic fever in macaques^7^.

With the exception of simarteviruses, all arteriviruses share a similar genomic organization. The arterivirus genome typically is a single 12–16 kb polyadenylated RNA that can be divided into two major regions. The 5′ region contains open reading frames (ORFs) 1a and 1b, which, through a combination of ribosomal frameshifting and subsequent proteolytic cleavage of the resulting polyprotein, give rise to the viral polymerase and other non-structural proteins^2,8^. The 3′ region encodes the structural components of the virion. Eight ORFs encode the envelope protein (E), five glycoproteins (GP2, GP3, GP4, GP5, and GP5a), the membrane protein (M), and the nucleocapsid protein (N). Aside from the function of the proteins they encode, the two regions also differ regarding the mechanisms used for protein expression. Whereas the virus polyprotein is translated directly from the genomic RNA, the 3′ region of the genome is used as a template for the generation of a nested set of subgenomic negative-sense RNAs, from which subgenomic (positive-sense) mRNAs encoding the different structural proteins are transcribed. Unlike other arteriviruses, simartevirus genomes contain a large insertion, located upstream of ORF2a, that encodes an additional three or four structural proteins^3,9^. Although the function of these additional proteins remains to be fully elucidated, they have been shown to play an important role in the production of infectious virions^10^. Furthermore, recent research investigating the coding capacity of the SHFV genome has revealed the presence of numerous additional ORFs, showing that the transcriptional organization of arterivirus genomes may be more complex than hitherto assumed^11^.

Here we describe the complete genome sequences of three variants of a novel arterivirus, Olivier’s shrew virus 1 (OSV-1), detected in the serum of Guinean Olivier’s shrews (*Crocidura olivieri guineensis*). Our analyses indicate that this arterivirus, the first to be discovered in members of the order Eulipotyphla, represents a member of a new arterivirus genus.

## Results

### Discovery of a novel arterivirus

In this study, we chose to screen four Olivier’s shrews (Shrews 1–4), caught and killed near Guéckédou, Guinea, for any potential viruses they might harbor. None of the animals displayed macroscopic signs of disease at the time of their capture (tissues were not examined for pathology due to field safety concerns). RNA, extracted from the serum of Shrew 1, was subjected to nanopore sequencing, revealing the presence of a previously undescribed arterivirus. Subsequently, Illumina sequencing was performed on the RNA extracted from Shrew 1 to further correct the arteriviral genome sequence obtained through nanopore sequencing. Illumina sequencing was also performed on serum RNA from Shrews 2–4 to test if these animals harbored the same virus.

Three out of four serum samples contained arterivirus sequences. For two of the samples (Shrews 1 and 2), Illumina sequencing yielded sufficient data to determine the coding-complete genome sequence of the present viruses. In the case of Shrew 1, the sequence similarity between the newly generated contig (Illumina sequencing only) and the corrected nanopore contig (Nanopore + Illumina sequencing) was 100%. In the case of Shrew 3, insufficient viral data was obtained to assemble a single, full-length contig. Sanger sequencing was therefore used to complete the viral genome sequence. The serum from Shrew 4 did not contain any arteriviral sequences. In addition to serum, arterivirus RNA could also be detected in harvested shrew heart, kidney, liver, lung, and spleen samples of Shrews 1 and 3. In Shrew 2, the virus was only found in serum. Rapid Amplification of 5′ cDNA ends (5′ RACE) was used on sera from Shrews 1–3 to complete the arterivirus genome sequences. The completed genomes are 13,766 (Shrew 1: variant A, GenBank: MF324848), 13,760 (Shrew 2: variant B, GenBank: MF324849), and 13,763 nucleotides (Shrew 3: variant C GenBank: MG264317) long and share 88–89% similarity on the nucleotide level. This similarity indicates that the three viruses represent different variants of the same virus, here named Olivier’s shrew virus 1 (OSV-1).

### Genome organization of Olivier’s shrew virus 1

**Figure 1:**
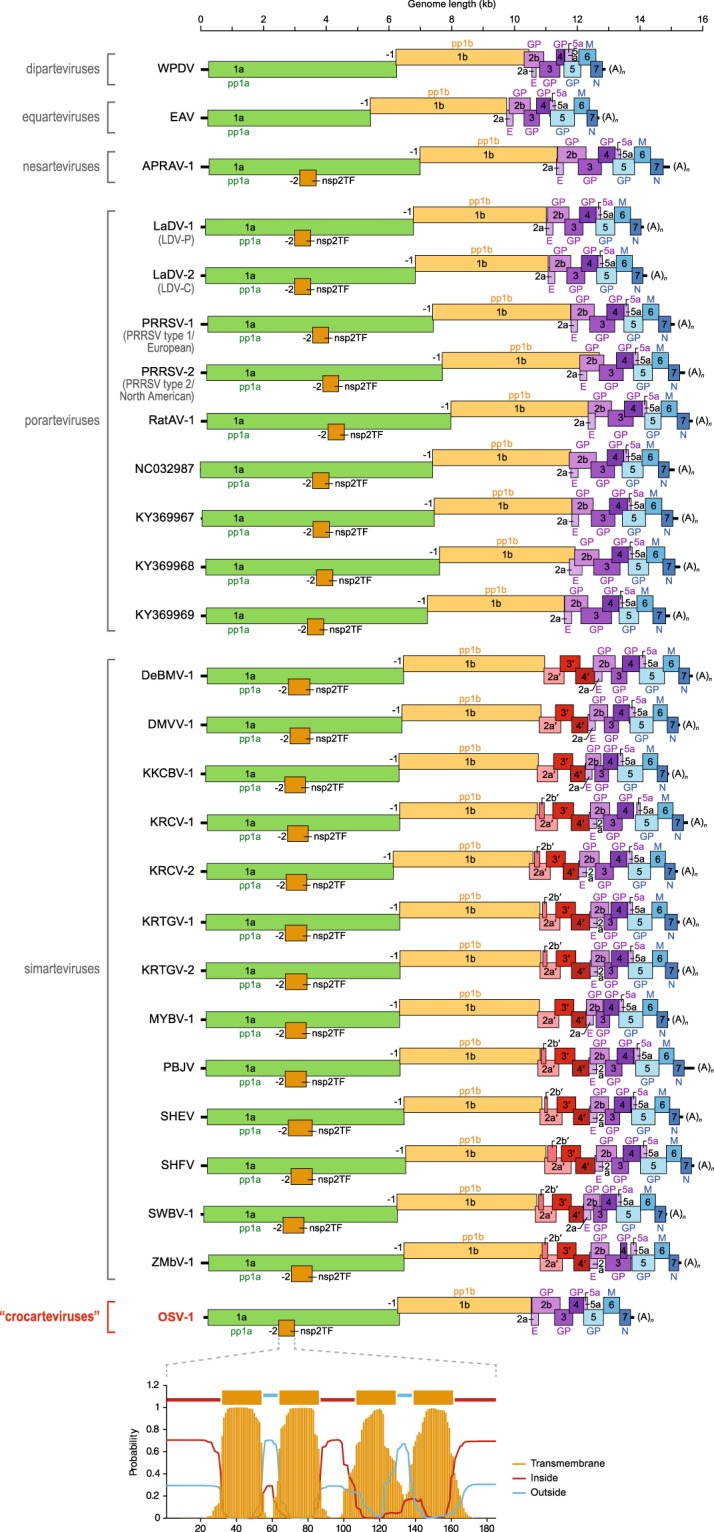
Organization of arterivirus genomes (updated from^3^). Arterivirus open reading frames (ORFs) are drawn to scale. ORFs 1a and 1b can be joined through a −1 programmed ribosomal frameshift to express polyprotein 1a,b. Nonstructural protein 2 transframe fusion product (nsp2TF) is produced through a −2 programmed ribosomal frameshift by most, but not all, arteriviruses. The plot at the bottom of the figure shows the predicted organization of this TF as a transmembrane domain in the case of OSV-1. This plot was made using TMHMM server v2.0 (http://www.cbs.dtu.dk/services/TMHMM/). WPDV, wobbly possum disease virus (GenBank #JN116253); EAV, equine arteritis virus (NC_002532); APRAV-1, African pouched rat virus 1 (NC_026439.1); LaDV-1/2, lactate dehydrogenase-elevating virus 1 and 2 (NC_001639 and L13298.1); PRRSV-1/2, porcine reproductive and respiratory syndrome viruses 1 and 2 (M96262 and NC_001961); RatAV-1, rat arterivirus 1 (NC028963); DeBMV-1, DeBrazza’s monkey virus 1 (NC_026509); DMVV-1; Drakensberg Mountain vervet virus 1 (NC_029992); KKCBV-1, Kafue kinda-chacma baboon virus 1 (NC029053); KRCV-1/2, Kibale red colobus viruses 1 and 2 (HQ845737 and KC787631.1); KRTGV-1/2, Kibale red-tailed guenon viruses 1 and 2 (JX473849 and JX473847); MYBV-1, Mikumi yellow baboon virus 1 (NC_025112.1); PBJV, Pebjah virus (KR139838); SHEV, simian hemorrhagic encephalitis virus (KM677927); SHFV, simian hemorrhagic fever virus (NC_003092); SWBV-1, Southwest baboon virus 1 (NC_025113.1); ZMbV-1, Zambian malbrouck virus 1 (KT166441); OSV-1, Olivier’s shrew virus 1 (MF324848). Four additional murid porarteviruses that have not yet been described are identified by GenBank accession numbers only.

The three OSV-1 genomes contain the ten arterivirus-typical ORFs 1a/1b, 2a, 2b, 3, 4, 5, 5a, 6, and 7 that encode the arterivirus (poly)proteins pp1a/pp1ab, E, GP2, GP3, GP4, GP5, GP5a, M, and N, respectively (Fig. 1)^2,3,8^. In all previously described arteriviruses, pp1ab is expressed via a −1 programmed ribosomal frameshift that joins ORF 1a with the overlapping ORF 1b^12^. The frameshift site typically presents itself as a ‘slippery sequence’ of the form X_XXY_YYZ (with XXX being any trinucleotide sequence, YYY being AAA or UUU and Z being an A, T, or C), located a few nucleotides upstream of a stable RNA secondary structure^8^. This motif, starting with the putative −1 frameshift site, was also found in OSV-1 (U_UUA_AAC at positions 6,220–6,226, 6,214–6,220, and 6,217–6,223 for variants A–C, respectively).

In addition to the generation of the large polyprotein (pp)1ab, several arteriviruses also use −1 and −2 programmed ribosomal frameshifting to produce, respectively, a truncated nonstructural protein 2 (nsp2), ‘nsp2N’, and an nsp2-transframe fusion product (‘nsp2TF’)^13^. The arterivirus *nsp2* gene is a multidomain gene that encodes a protein, which, amongst other functions, acts as a proteinase and is involved in the formation of replication complexes^14^. The −2-ribosomal frameshift within this gene results in the replacement of the two 3′-terminal domains, a transmembrane domain and a cysteine-rich region, by a different transmembrane domain encoded in an alternative reading frame in the nsp2 region of ORF1a. Knockout of nsp2TF is known to result in reduced viral fitness, and recent work demonstrated that both nsp2TF and nsp2N are involved in suppressing host innate immune responses during PRRSV-1/2 infections^15,16^. In the case of PRRSV-1/2, this −1/−2 frameshifting occurs at a conserved G_GUU_UUU motif, followed by a CCCANCUCC motif a few nucleotides downstream^15^. Minor variants of these two motifs can be found in the genomes of all arteriviruses with the exception of equarteviruses (EAV)^17^. In the genomes of OSV-1 variants A–C, slightly altered versions of these two motifs are present (G_GUU_UUC, CCCCGGUCC) in the vicinity of a TF encoding a transmembrane domain (Fig. 1 bottom). This TF is located approximately at the same position as the TF found in other arteriviruses (overlapping the nsp2 transmembrane domain). Whether or not it is functional remains to be experimentally established.

Besides the ORF 1a- and 1b-derived pp1a and pp1ab, the OSV-1 genome also encodes at least eight smaller, structural proteins, including the arterivirus-typical E, M and N proteins and glycoproteins GP2–GP5 and GP5a. The additional set of ORFs characteristic of simartevirus genomes (ORFs 2a′, 2b′, 3′, and 4′) is not present in the OSV-1 genome.

### Phylogenetic analysis of Olivier’s shrew virus 1

**Figure 2:**
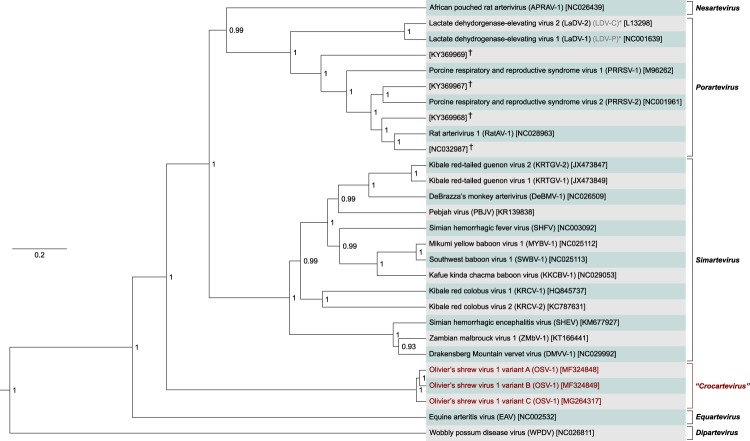
Maximum clade credibility tree of arteriviruses representing all currently known species based on the sequence of ORF 1b. Phylogenetic inference using Bayesian phylogenetics shows that OSV-1 variants A–C (marked in red) cluster together but are separate from other arteriviruses. The numbers at the different nodes indicate the posterior probabilities of the accuracy of loci, given the used data and chosen model and parameters. The tree is drawn to scale, with branch lengths representing the number of substitutions per site. GenBank accession numbers of all used sequences are included in brackets. *Previous designation (C/P) of lactate dehydrogenase-elevating virus strains 2/1. ^†^As of now, these viruses have not yet been described and are known only as ‘rat/rodent arterivirus(es)’.

**Figure 3:**
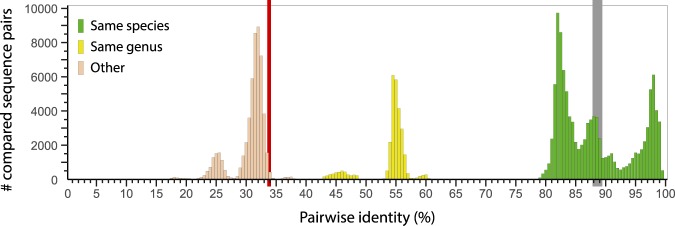
Pairwise sequence comparison analysis of arterivirus genomes, including the newly discovered three complete genome sequences of Olivier’s shrew virus 1 (OSV-1) variants A–C, using the NCBI PASC tool (https://www.ncbi.nlm.nih.gov/sutils/pasc)^18^. Pairwise similarity between the three sequences ranges from 87.89–89.39% (marked in grey), indicating all three sequences belong to viruses that ought to be classified in the same species. Pairwise similarity with the closest related virus, porcine reproductive and respiratory syndrome virus 1 (PRRSV-1), ranges from 33.49–34.01% (marked in red), indicating the need for the establishment of a new arterivirus genus and species. Color coding was modified to accurately represent the current taxonomic organization of the family *Arteriviridae*, using 39–41% and 71–77% as genus and species cut-offs, respectively.

Bayesian phylogenetic analysis of representative genome sequences of all known arteriviruses using the deduced ORF 1b amino acid sequences groups OSV-1 variants A–C together in a distinct lineage within the family *Arteriviridae* (Fig. 2). Pairwise sequence comparison of the complete OSV-1 variants A–C genomes, using the US National Center for Biotechnology Information (NCBI) PAirwise Sequence Comparison (PASC) tool^18^, shows 87.89–89.39% pairwise identity between the three variants and identifies PRRSV-1 as the closest related virus. OSV-1 variant A is most closely related to PRRSV-1 isolate CReSA70 (GenBank: KX249752), whereas variants B and C are most closely related to PRRSV-1 isolate CReSA46 (GenBank: KX249751). The pairwise identities are 34.01% (variant A), 33.49% (variant B), and 33.92% (variant C), respectively (Fig. 3). The latest accepted proposal of the International Committee on Taxonomy of Viruses (ICTV) for arterivirus classification mandates 39–41% and 71–77% pairwise identities to be the most appropriate genus and species demarcation cut-offs^3,19^. As both phylogenetic inference and pairwise sequence comparison indicate that OSV-1 clearly diverges from established arterivirus genera, we propose the creation of a new genus and species to which OSV-1 can be assigned.

## Discussion

We tested four Olivier’s shrews caught and killed in Guinea for potential infection with previously unidentified viruses. Serum from three of these animals tested positive for a yet undescribed arterivirus. Using a combination of different sequencing methods, we determined the complete genome sequence of three variants of this virus, which was given the name Olivier’s shrew virus 1 (OSV-1). As illustrated by both PASC and phylogenetic inference, OSV-1 strongly diverges from other, previously known arteriviruses, warranting the establishment of a new genus within the family *Arteriviridae*.

The current nomenclature of arterivirus genera (*Dipartevirus*, *Equartevirus*, *Nesartevirus*, *Porartevirus*, *Simartevirus*) is based on the contraction of a short prefix referring to the viruses’ hosts (Diprodontia, Equidae, Nesomyidae, porcine and rodent, and simian, respectively), and the suffix -*artevirus* (a contraction of *Arteriviru*s)^3,19^. Following this guidance, we propose that OSV-1 be assigned to a new genus, named *Crocartevirus* (Oliver’s shrews are members of the soricid subfamily Crocidurinae).

Prior to euthanasia, all shrews screened during this study appeared to be in good health and had no macroscopic signs of disease. In two shrews, OSV-1 was found in all tested organs (heart, liver, lung, kidney, and spleen) in addition to serum, indicating systemic subclinical infection. In the third shrew, OSV-1 was detected only in the serum. A potential explanation for this could be that infection was still in an early phase, and the virus had not yet disseminated to different organs. Whether infections of Olivier’s shrews with OSV-1 remain subclinical or can give rise to overt disease remains to be established.

Since we only tested four shrews, speculation on the general distribution of OSV-1 in shrew populations is difficult. The animals studied here were caught over the course of a week in three different locations within a 4-km radius. Because of the distance between the different trapping locations, albeit limited, animals were unlikely to infect each other. This assertion is also corroborated by the substantial sequence divergence between the different virus variants identified here (>10%). Furthermore, as three out of four shrews tested harbored the virus, a reasonably high prevalence is conceivable. Determining the host range of OSV-1 is of interest, as it is possible that the virus can infect crocidurine shrews other than Olivier’s shrews. Alternatively, as OSV-1 represents the first eulipotyphlan arterivirus, a wide variety of distinct arteriviruses could exist in shrews and their relatives (e.g., desmans, hedgehogs, moles, moonrats, and shrew moles). In fact, the relatively recent discovery of WPDV in Australian brushtail possums (*Trichosurus vulpecula*) in New Zealand^6^ and this discovery of OSV-1 suggest the possibility that arteriviruses infect animals of all mammal branches globally. In addition, induction of overt disease may be the exception rather than the rule.

## Materials and Methods

### Ethics statement

This study was approved and supervised by the KU Leuven Animal Welfare Body (KU Leuven LA1210186) in compliance with Guinean, Belgian and European statutes and regulations relating to animals and experiments involving animals.

### Sample collection

For routine pest control in a domestic setting, small animals were trapped and killed in February 2016 near Guéckédou (Nzérékoré Region, Guinea). Among the killed mammals were four animals thought to be African giant shrews (Eulipotyphla: Soricidae: Crocidurinae: *Crocidura olivieri*), based on a close examination of their external features. This initial assessment was confirmed by sequencing the cytochrome B gene of these animals and comparing the resulting sequences to known shrew cytochrome B genes using blastn (data not shown)^20^. Several organs (hearts, lungs, spleens, kidneys, and livers) and serum were collected from all four animals (Shrews 1–4) for further analysis.

### Virus genome sequencing

Collected organs and serum were subjected to viral RNA extraction using the RNeasy Mini kit (QIAGEN Benelux, Venlo, Netherlands) according to the manufacturer’s instructions. No infections or work with infectious viruses was performed. Consequently, all experiments after initial animal capture and processing in the field was performed at biosafety level 1. Serum RNA from Shrew 1 was selected for nanopore sequencing using the Oxford Nanopore MinION (Oxford Nanopore Technologies, Oxford, UK). The extracted RNA was amplified by whole-transcriptome amplification (WTA2; Sigma-Aldrich, St. Louis, MS, USA), and the resulting WTA product was used in combination with the SQK-LSK108 kit (Oxford Nanopore Technologies) to prepare the MinION sequencing library. A total of 244 ng of cDNA was prepared for sequencing according to the manufacturer’s ‘1D genomic DNA by ligation’ protocol with the omission of the initial DNA-shearing step. The resulting sequencing library was loaded onto a R9.4 flow cell supplied by the manufacturer and run for 7.5 h. Non-viral reads were filtered out with the use of tblastx^20^. The remaining reads were assembled using Canu^21^, resulting in a single 13-kb contig.

In addition, total RNA extracted from all sera (Shrews 1–4) was amplified by whole-transcriptome amplification (WTA2; Sigma-Aldrich). The resulting cDNAs were prepared for Illumina NextSeq 500 sequencing (Illumina, Hayward, CA, US) using the Nextera XT DNA library preparation kit (Illumina), according to the manufacturer’s instructions. De novo assemblies of the generated data were made using CLC Genomics Workbench (v10.0.1; QIAGEN). DIAMOND was used to identify the resulting contigs^22^. Illumina data for Shrew 1 were used to further correct the Shrew 1 contig previously obtained by nanopore sequencing.

Due to the limited yield of arterivirus sequence reads in the case of Shrew 3 using the Illumina approach, we decided to use Sanger sequencing for genome sequence completion. We developed a set of degenerate primers based on the genomes of the arteriviruses found in Shrews 1 and 2. This primer set was used in combination with the OneStep RT-PCR kit (QIAGEN) to amplify the complete viral genome in a series of amplicons. The resulting PCR products were purified with ExoSAP-IT (Affymetrix, High Wycombe, UK) and prepared for sequencing using the BigDye Terminator v3.1 Cycle Sequencing Kit (Applied Biosystems, Carlsbad, CA). All Sanger sequencing was performed on an ABI Prism 3130xl Genetic Analyzer (Thermo Fisher Scientific, Waltham, MA, US). Open-source Chromas (v2.6.2, Technelysium, South Brisbane, AU) was used to inspect the resulting chromatogram files. In a second sequencing round, the sequences obtained from these amplicons were used to generate new, complementary primer sets to replace the degenerate primer sets that failed to generate amplicons. Finally, Seqman (v7.0.0, Madison, WI) was used to join all amplicon sequences. All used primer set sequences and PCR conditions are available on request.

### Determination of terminal virus genome sequences

The 5′/3′ RACE kit 2nd generation (Roche, Mannheim, DE) was used to generate poly-A-tailed cDNAs of genomic 5′ ends. These poly-A-tailed cDNAs were further amplified using the OneStep RT-PCR kit (QIAGEN) and the following PCR conditions: 15 min at 95 °C, 40 cycles of 30 s at 94 °C, 30 s at 56 °C, 1 min at 72 °C, and a final 10-min extension step at 72 °C. A second primer set was used to further amplify 2 µl of the resulting PCR product using the same reaction conditions (annealing temperature: 61 °C). Sanger sequencing of the obtained PCR products was performed as described above. After inspecting the chromatogram files using Chromas (v2.6.2), the resulting sequences were joined with the rest of their respective genome sequences using Seqman (v7.0.0).

### Phylogenetic analysis

Deduced ORF 1b amino acid sequences of all known arteriviruses were aligned using the Multiple Alignment Program for Amino Acid or Nucleotide Sequences (MAFFT, v7.123b)^23^. After trimming with trimAL (1.2rev59)^24^, the resulting multiple sequence alignments were manually edited in MEGA7^25^. Bayesian phylogenetic trees were inferred with Bayesian Evolutionary Analysis by Sampling Trees (BEAST) 2 using a Whelan Goldman (WAG) model to describe the amino acid substitution process^26^. The Markov chain Monte Carlo analyses were run until adequate effective sample sizes (ESS > 200) were obtained. A maximum clade credibility tree was summarized from the posterior tree distribution with TreeAnnotator (v2.5.4) using a burn-in of 10%, and visualized with FigTree (v1.4.3)^26^. The NCBI PASC tool^18^ was used to assess the classification of the discovered arterivirus variants within the family *Arteriviridae*.

### Data availability

All data generated during this study are included in this published article or in the referenced GenBank entries.
